# Development of a Mobile Health Intervention to Promote Papanicolaou Tests and Human Papillomavirus Vaccination in an Underserved Immigrant Population: A Culturally Targeted and Individually Tailored Text Messaging Approach

**DOI:** 10.2196/13256

**Published:** 2019-06-06

**Authors:** Hee Yun Lee, Mi Hwa Lee, Monica Sharratt, Sohye Lee, Anne Blaes

**Affiliations:** 1 School of Social Work The University of Alabama Tuscaloosa, AL United States; 2 School of Social Work College of Heath and Human Performance East Carolina University Greenville, NC United States; 3 School of Social Work College of Education and Human Development University of Minnesota Twin Cities, MN United States; 4 Loewenberg College of Nursing University of Memphis Memphis, TN United States; 5 Division of Hematology, Oncology and Transplantation Department of Medicine University of Minnesota Twin Cities, MN United States

**Keywords:** uterine cervical cancer, papanicolaou test, papillomavirus infections, papillomavirus vaccines, text messaging, Asian American, immigrants

## Abstract

**Background:**

Disparities in cervical cancer incidence and mortality signify the need for intervention efforts targeting Korean American immigrant women.

**Objective:**

The purpose of this study was to demonstrate how a culturally targeted and tailored mobile text messaging intervention, mobile screening (mScreening), was developed to promote the uptake of Papanicolaou tests and human papillomavirus vaccine among young Korean American immigrant women.

**Methods:**

Guided by the Fogg behavior model, the mScreening intervention was developed through a series of focus groups. Braun and Clarke’s thematic analysis was used to identify core themes.

**Results:**

Overall, 4 themes were identified: (1) tailored message content (ie, basic knowledge about cervical cancer), (2) an interactive and visual message format (ie, age-appropriate and friendly messages using emoticons), (3) brief message delivery formats to promote participant engagement, and (4) use of an incentive to motivate participation (ie, gift cards).

**Conclusions:**

This study demonstrated the processes of gathering culturally relevant information to develop a mobile phone text messaging intervention and incorporating the target population’s perspectives into the development of the intervention. The findings of the study could help guide future intervention development targeting different types of cancer screening in other underserved racial or ethnic groups.

## Introduction

### Background

Although cervical cancer has become a largely preventable disease with recent reductions in cervical cancer mortality in the United States [1], it still remains a major concern in the field of women’s health. Approximately 12,000 women are newly diagnosed with cervical cancer and 4000 mortalities occur annually [2]. Although there is lower overall cervical cancer incidence and mortality across the aggregate Asian American group when compared with other ethnic or racial groups [3], certain Asian subgroups report some of the highest rates of cervical cancer across all ethnic or racial groups. For example, Korean American immigrant women were found to have an incidence rate of 11.9 per 100,000, much higher in contrast with non-Latino whites (7.1), Japanese (6.2), and Chinese (5.8) samples [4].

The Papanicolaou (Pap) test is a screening procedure for cervical cancer that helps to facilitate earlier detection and treatment. The US Preventive Services Task Force recommends a Pap test for all women between the ages of 21 and 65 years every 3 years [5]. Although 72.8% of non-Latino white women aged 18 years and older had reported completing a Pap test within the past 3 years in 2010, only 68.0% of Asian women had done the same [6]. One potential reason behind the disproportionate burden of cervical cancer faced by Korean American immigrant women might be the low uptake of both the Pap tests and the human papillomavirus (HPV) vaccines. In accordance with that disparity, Korean American immigrant women have consistently demonstrated a much lower likelihood of being up to date with recommended Pap tests than non-Latina white women [7,8]. A previous study in California reported that 35% of Korean American immigrant women aged 18 to 65 years have not received a Pap test within the past 3 years [9].

The HPV vaccine prevents infection from the strains of HPV that cause cervical cancer. The HPV vaccine was introduced in 2006 for women and in 2008 for men and is recommended for all adolescents starting at the age of 9 years, as well as young women and men up to the age of 26 years [10]. Despite the availability of the HPV vaccine, an effective mechanism for preventing cervical cancer, the rate of vaccination in Asian American immigrant women is significantly lower (38.6%) than in non-Latina white women (60.7%) [11]. For example, only 24% of Korean American immigrant mothers reported that their vaccine-eligible daughters had initiated the HPV vaccine series of 3 doses [12]. Therefore, an effective intervention is needed to promote HPV vaccination to prevent cervical cancer for Korean American immigrant populations.

The disparities in utilization of the Pap tests and HPV vaccine highlight the need for intervention efforts to specifically target Korean American immigrant women. Previous efforts to reduce cervical cancer were focused on promoting Pap tests in Asian American women, such as community-based navigation [13], lay health workers [14], and lay health worker outreach combined with media intervention [15]. However, these studies reported mixed outcomes in improving Pap testing. Additional interventions to promote Pap tests and HPV vaccines specifically targeting underserved women such as Korean American immigrant women are needed [16-18].

Incorporating mobile health (mHealth) technology into these interventions might improve outcomes. Text messaging has previously been shown to be a useful and efficient means of educating patients on sensitive health-related issues that require confidentiality and security, such as HIV prevention [19], mental health [20], sexually transmitted infection management [21], and smoking cessation [22-24]. As such, it is possible that the incorporation of mHealth technology could help improve the efficacy of efforts to promote Pap tests and HPV vaccination.

### Objectives

Although mobile phone–based interventions appear to hold promise as effective vehicles promoting behavioral change, little knowledge has been established regarding how to successfully develop such intervention programs for cervical cancer prevention. This study aimed to illustrate how a culturally targeted and individually tailored mobile text messaging intervention, mobile screening (*mScreening*), was developed. The *mScreening* is a 7-day educational program to promote the uptake of the Pap tests and HPV vaccines among young Korean American immigrant women aged between 21 and 29 years. Our *mScreening* studies showed the effectiveness and feasibility of the intervention, including improved knowledge of cervical cancer and Pap test rates [25] and raising knowledge, attitude, and uptake rates of HPV vaccine [26]. This study retrospectively shares the design methodology based on the success we had with the solution.

We targeted young Korean American immigrant women aged between 21 and 29 years because of age guidelines of receipt of Pap tests (21 to 65 years) and HPV vaccines (18 to 26 years). Given that young Korean American immigrant women have a high accessibility to mobile phones [27], we targeted young Korean American immigrant women as an intervention group. We also targeted both the Pap test and the HPV vaccine as primary outcomes given that they are highly important prevention strategies for cervical cancer, with the Pap test helping to facilitate earlier detection and treatment of cervical cancer and the HPV vaccine helping to reduce the likelihood of cervical cancer.

## Methods

### Conceptual Framework

The procedure for developing *mScreening* was guided by the Fogg behavior model (FBM) [28]. The FBM consists of 3 components: identifying barriers, developing motivators, and providing triggers to act. This theory basically posits that to make positive health behavior happen, researchers should identify barriers to a specific health behavior, convert the barriers to motivators, and finally, provide triggers to make the action occur.

**Figure 1:**
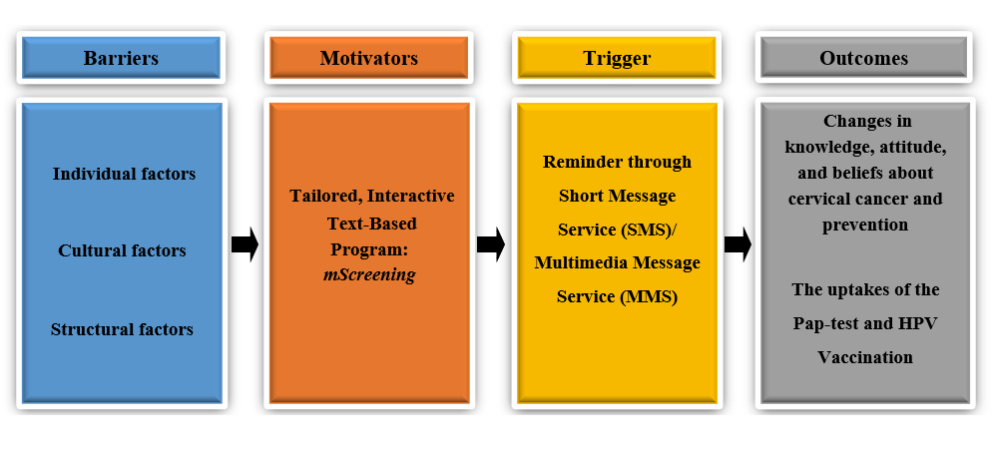
Conceptual framework.

Figure 1 shows the specific approach that we proposed using FBM. To identify the barriers and develop motivators and triggers for Pap test uptake and HPV vaccine receipt in young Korean American immigrant women, a series of focus groups was conducted. Through the focus groups, 3 barriers were identified and previously published, including limited knowledge about cervical cancer and preventive behaviors, cultural barriers, and low accessibility to health care services [29]. On the basis of these identified barriers, motivators and triggers were also developed.

In addition to the focus group meetings, the research team had regular meetings with community advisory board members and mobile technology developers to get feedback and refine the *mScreening.* Through periodic meetings, strategies and topics—such as what messages and pictures would motivate young Korean American immigrant women, what real life stories exist, how the stories should be presented, and in what format the content should be delivered–were discussed and finalized. The community advisory board consisted of Korean American immigrant women in their 20s, a Korean American physician, a Korean American nurse, and religious and community leaders. Furthermore, 2 mobile phone technology experts also reviewed the developed texting program and helped the research team to revise and finalize it.

### Research Design and Sampling

As mentioned earlier, we conducted a series of focus groups with young Korean American immigrant women to identify their barriers and to develop motivators and triggers for the use of Pap tests and HPV vaccine receipt. Overall, 5 focus groups were conducted between April 2011 and March 2012 with a total of 22 young Korean American immigrant women residing in the Twin Cities area of Minnesota, and the details of the procedure were published previously [25]. A total of 5 women participated in the first focus group, 7 in the second group, 4 in the third group, 3 in the fourth group, and 3 in the fifth group. In addition, 2 women participated twice; thus, we only count 20 women for the total number. The participants were recruited from postings on Korean American student associations’ websites, flyers at Korean churches, and the research team members’ personal networks. They were able to attend the focus groups regardless of their Pap test or HPV vaccine history. Each participant received US $20 for their time commitment.

### Data Collection

All of the focus groups were conducted in Korean and lasted approximately 1.5 to 2 hours. The first author (HL), along with 2 research staff, facilitated each focus group. The first author started the focus groups with welcoming messages, used ice breakers to build a rapport (eg, talking about the weather), and introduced the purpose of the meeting. The first author mainly asked questions to the participants and the other 2 research staff supported the first authors by asking follow-up questions and taking notes. The participants were informed about confidentiality, and they had a chance to introduce themselves to the group. Verbal and written consent was obtained from all participants before starting the groups. The topics of the focus groups included (1) barriers and facilitators of cervical cancer screening and HPV vaccination, (2) effective ways to increase Korean American immigrant women’s awareness of cervical cancer screening and HPV vaccination, (3) patterns of mobile phone use and ways to use mobile phone text messaging as an intervention medium to increase Pap testing and HPV vaccination, (4) ways to effectively deliver messages and age-appropriate designs for the intervention, and (5) potential implementation methods that would maximize participation and engagement. All sessions were digitally recorded to ensure that no information was missed during the sessions. Topic 1 (barriers to cervical cancer screening and HPV vaccination) was published [29]. This study reports on topics 2 to 5. The University of Minnesota institutional review board approved this study.

### Data Analysis

Core themes from the focus groups were identified using Braun and Clarke’s thematic qualitative method of analysis [30]. This method involves 6 phases: (1) becoming familiar with the data through transcription of verbal data, (2) generating initial codes, (3) searching for themes, (4) reviewing themes, (5) defining and naming themes, and (6) producing the report. For the first phase, all recordings were transcribed by a bilingual Korean research assistant and then reviewed by the research team. For the second phase, to make sure that no particular framework was imposed, the transcripts were coded by 3 different Korean bilingual researchers. Each researcher identified and highlighted every codable unit of text in the transcripts, then compared their analyses and agreed upon a set of codes, subcategories, and categories. Subsequently, based on the generated codes, the research team identified and reviewed themes present in the transcripts. Themes were compared across each transcript to ensure that they were representative and inclusive of all transcripts. Through this process, clear definitions and names for each theme were generated. Finally, the most representative quotes were selected to present in this paper and translated into English. For the translation process, a bilingual research assistant first translated the quotes into English and then another bilingual research team member back-translated them into Korean to ensure that the meaning was not lost in translation. All translations were then finalized by the first author.

## Results

### Study Participants

Table 1 presents the sociodemographic information of the focus group participants. The average age of the participants was 26 years, and 95% of the participants reported that they were unmarried. Most of the participants indicated that their families lived in Korea, and all participants were born and raised in Korea. They were undergraduate or graduate students in Minnesota at the time of this study. Participants reported majoring in various disciplines, including economics, biology, global studies, psychology, music, English literature, and social work. The average length of time they had resided in the United States was 3.9 years. All participants had health insurance at the time of the study.

### Themes From Focus Groups

Overall, 4 themes were identified from the focus group data about developing motivated messages to make health behavior change: (1) culturally targeted and tailored messages, (2) an interactive and visually appealing message format, (3) brief message delivery formats to promote participant engagement, and (4) the use of an incentive to motivate participation. In the rest of this section, each theme will be described and how it informed the development of *mScreening* will be explained. To protect the confidentiality of each participant, we only described each participant’s number in the study when presenting quotes.

#### Theme 1: Culturally Targeted and Individually Tailored Messages

Participants agreed on the importance of disseminating specific information on cervical cancer and ways to detect or prevent it to young Korean American immigrant women. The participants suggested that the following 3 content areas should be included and tailored when disseminating knowledge: (1) tailored statistics about cervical cancer incidence and mortality to raise awareness of the severity of cervical cancer, (2) culturally targeted message development to deliver knowledge of what the Pap tests and HPV vaccine are, and (3) tailored content to improve knowledge related to health care access on how to get the Pap tests and HPV vaccine.

**Table 1:** Sociodemographic characteristics of the sample (n=20).

Categories		Frequency, n (%)
**Age (years; mean=26 years)**		
	20-24	10 (40)
	25-29	12 (25)
**Marital status**		
	Unmarried	19 (95)
	Married	1 (5)
**Education^a^**		
	Undergraduate	13 (65)
	Graduate	7 (35)
**Years in the US (mean=3.9 years)**		
	Less than 5 years	13 (65)
	More than 5 years	7 (35)
**Health insurance**		
	Yes	20 (100)
	No	0 (0)

^a^Majors: biology, economics, English literature, global studies, music, psychology, and social work.

On the basis of the participants’ suggestions, a 7-day text message program was developed. First, *mScreening* introduced information about cervical cancer. Then, the Pap test was explained, followed by information on clinics and health professionals, as well as the cost of the Pap test. Next, HPV and the HPV vaccine were introduced and described, followed by information on the cost of the HPV vaccine and possible cultural barriers. The last day consisted of summarizing what they have learned through the 6 days using quizzes and games. The *mScreening* messages for each content area were developed using the message-framing techniques that affect health decision making based on the FBM model [28]. The FBM model particularly emphasizes that to make positive health behavior occur, barriers to specific health behavior (eg, Pap test and HPV vaccine) should be converted to motivators and triggers to action.

##### Subtheme 1.1: Tailored Statistics About Cervical Cancer Incidence and Mortality to Raise Awareness of the Severity of Cervical Cancer

One of the main content areas identified was the importance of educating the target population to improve their limited knowledge of and indifference to the topic of cervical cancer prevention. Participants expressed their desire to learn the definition of cervical cancer, risk factors for cervical cancer, and the incidence and mortality rates of cervical cancer among young women by race and ethnicity. In addition, they suggested that some of the information should be presented in a way that is *shocking* or *surprising*:

A shocking method! Young people are not easily shocked. Provide shocking information...something that can alert the young people to think that it is possible for them to have cervical cancer.

Participant #15

**Figure 2:**
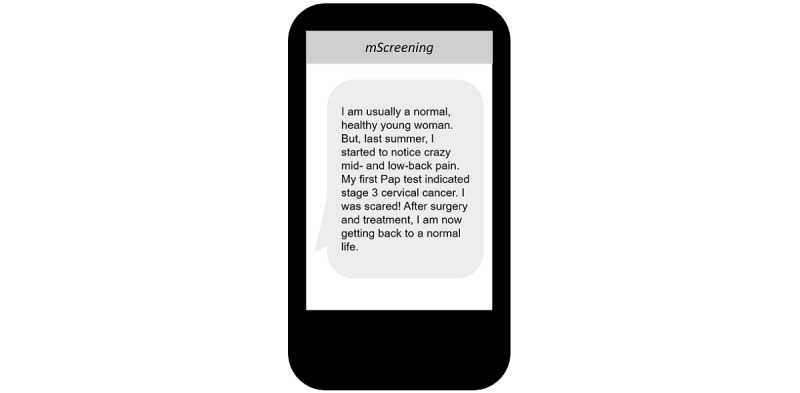
Mobile screening message example: used a testimony from a young Korean American immigrant woman.

The research team sought to integrate participants’ suggestions about creating shocking or surprising messages on the topic of basic knowledge about cervical cancer to capture the target population’s attention, thereby increasing their awareness. For example, the messages included images that compared a healthy cervix with an unhealthy cervix, statistics on mortality, incidence, and screening rates for cervical cancer in Korean American immigrant women compared with other racial or ethnic groups, and testimony from a young Korean American immigrant woman who was diagnosed with cervical cancer (Figure 2).

##### Subtheme 1.2: Culturally Targeted Message Development to Deliver Knowledge of the Pap Tests and Human Papillomavirus Vaccine

The participants expressed expectations that the text messages would include information on the guidelines, process, cost, and pain level of Pap testing. They also advised that the messages should be targeted to revise cultural misconceptions about cervical cancer screening and prevention behaviors. Almost all the participants reported that they had not heard of cervical cancer or screening before the study or, even if they had, that they had paid little attention to the need for prevention. Interestingly, some participants stated that they wanted information related to the HPV vaccine and its side effects to be provided by a doctor because of a lack of trust they held toward other sources of vaccination information, which was the result of the excessive promotion of vaccinations via media in Korea.

**Figure 3:**
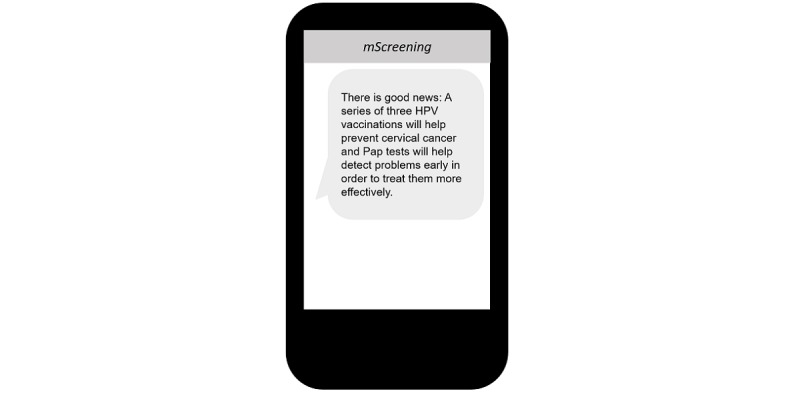
Mobile screening message example: focused on enhancing motivation.

As such, *mScreening* messages regarding the Pap tests and the HPV vaccine were developed focusing on enhancing motivation for carrying out cervical cancer prevention–related behaviors (Figure 3). For instance, messages were created to educate participants about the benefits of receiving the Pap test and HPV vaccination using a gain message frame [31]. This is one way of converting the barrier (lack of knowledge on Pap test and HPV and HPV vaccine) to motivator (empowering through education) to make positive health behavior change according to the FBM model [28]. These messages sought to promote Pap testing and HPV vaccine uptake by using a female doctor as the spokesperson. All participants suggested to use a female doctor to educate the content given that talking about women’s reproductive organs with a male doctor is embarrassing in Korean culture. Thus, it was decided that a female doctor would be a preferable spokesperson compared with a male doctor.

In addition, a majority of participants also expressed wanting text messages to be targeted to overcome cultural misinformation (eg, hymen breaking during a Pap test) and apprehension about cervical cancer screening (eg, feeling uncomfortable in a gynecology clinic as an unmarried female). The participants suggested several methods toward achieving this goal, such as including powerful emotional messages or life experience examples from people who have successfully overcome these obstacles:

Showing a real example...real person...like a student who got the shot can share what she thought about it before, what it really was like, and how she feels afterwards. That kind of example...[or] if a person who went to have [the Pap] test explains how she felt.

Participant #4

**Figure 4:**
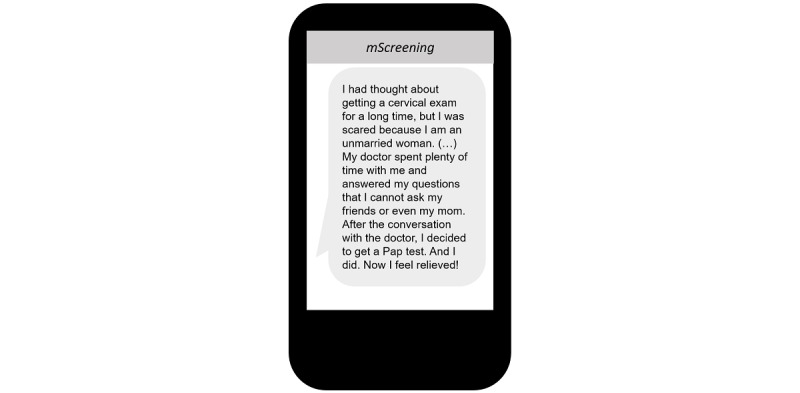
Mobile screening message example: reframed the Pap test more positively for young Korean American women.

To reflect the participants’ suggestions, testimonial text messages were created and incorporated into the *mScreening* by working with young Korean American immigrant women who had received the Pap test. Given that obstetric and gynecological health issues are culturally sensitive in the Korean American community, especially so for unmarried young women [32], the testimonial messages were designed to reframe the Pap test more positively for young Korean American women (Figure 4). In addition to being influenced by most of the participants’ suggestions, this approach is supported by previous research that suggests that sharing the personal experiences of individuals in the same ethnic group can be influential in changing participants’ cultural beliefs [33].

##### Subtheme 1.3: Tailored Content to Improve Knowledge Related to Health Care Access

Participants suggested including practical information about the costs of receiving the Pap test and HPV vaccination, clinics they could visit to receive these procedures, and how to make appointments in *mScreening.* Participants shared their views that practical information was needed because of their lack of knowledge about the US health care system:

I was surprised that the cost for the Pap test is free. [Korean] People may think medical costs are very high in the US system. They may go and get the screening if they know it is for anyone.

Participant #10

Participants also mentioned that it would be helpful to have messages from a doctor about the procedures related to Pap testing and HPV vaccination. Participants felt that this would increase their familiarity with the process and reduce their uneasiness. Furthermore, participants wanted to know about the availability of female doctors who performed Pap tests at the clinics because of their reluctance to engage in conversations about their periods or personal sexual activities with male doctors:

If I know that these [female] doctors are the ones that I meet with for an appointment, I would feel more comfortable making an appointment [to have a Pap test].

Participant #16

**Figure 5:**
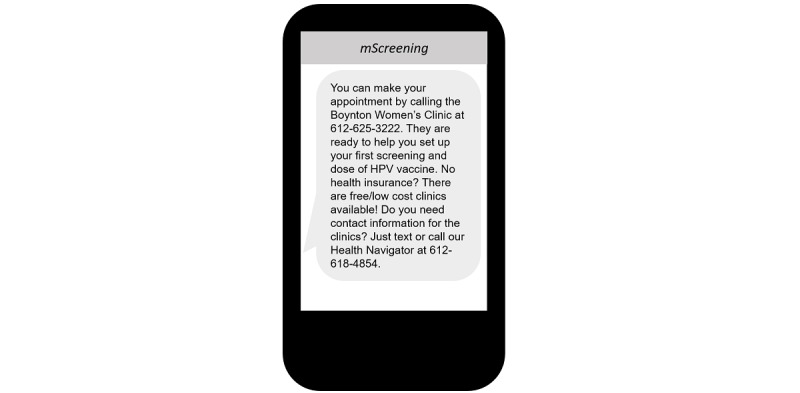
Mobile screening message example: included information on the price of the human papillomavirus vaccine and phone numbers of women’s clinics that employed female doctors and nurses.

As a result, the *mScreening* messages included information on the price of the HPV vaccine, phone numbers of women’s clinics that employed female doctors and nurses, and a list of available free or low-cost clinics in Minnesota. One example of a targeted message is shown in Figure 5.

#### Theme 2: Message Format—Interactive and Visually Appealing Messages

All participants agreed that mobile phones are a significant part of their daily lives. The participants’ endorsement of the high accessibility of mobile phones suggests that developing a texting program for the target population could be successful. Participants agreed with this stance when queried and suggested 3 types of messages that could be used together to maximize engagement: visual messages, interactive messages, and dynamic characters or emoticons. On the basis of this feedback, *mScreening* disseminated information using 3 types of messages as described below.

##### Subtheme 2.1: Visual Messages

Participants indicated a preference for visual messages that integrated video clips or images, such as a picture of a cervix and charts of mortality rates, over plain text:

Rather than texting, [it would be better to receive messages] through videos, images, posters, or cartoons because web cartoons are popular these days...

Participant #19

**Figure 6:**
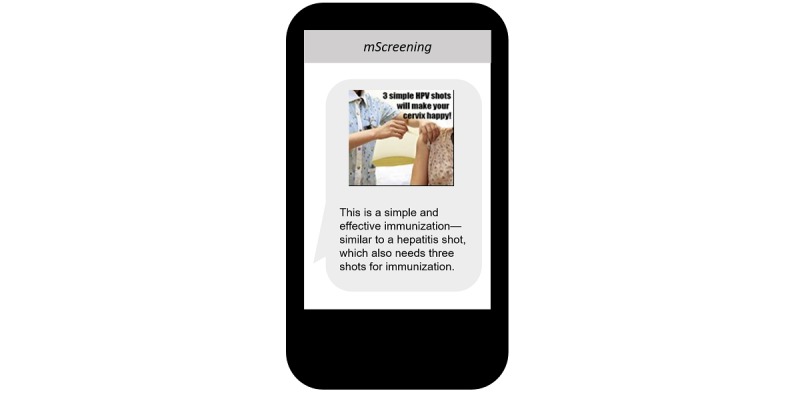
Mobile screening message example: demonstrated the power of visual messages to persuade people.

As a result of this input as well as previous studies that have demonstrated the power of visual messages to persuade people [34], *mScreening* included a variety of graphs, pictures, and drawings (Figure 6).

##### Subtheme 2.2: Young Age–Friendly Messages With Graphics

A majority of participants also highly recommended the inclusion of age-friendly messages that used characters or emoticons, stating that they would be more attractive and dynamic to the young Korean American population than simple texts, helping the participants feel more comfortable. As participants recommended, age-friendly messages with graphics (eg, emoticons, logos, and cartoons) were created to target young Korean American women. Given the fact that cervical cancer screening and prevention are not a common topic of discussion in the Korean American community, using age-appropriate text messages with cartoons was suggested as a way to culturally target the intervention and decrease discomfort around having conversations regarding screening and prevention:

If it is through a smartphone, it would be good to use cartoons or videos. This will keep me more focused and engaged [to learn cervical cancer screening and prevention methods.

Participant #15

**Figure 7:**
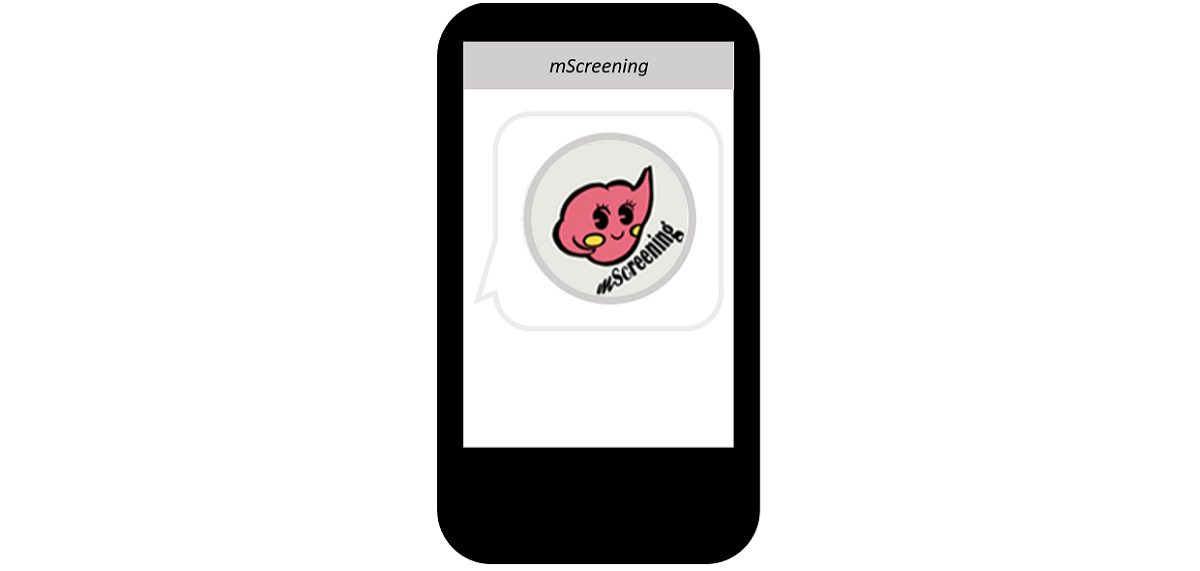
Mobile screening message example: created a study project logo.

To help achieve this suggestion, we first created the study’s logo, which symbolizes a happy and healthy cervix. The focus group participants provided multiple ideas of how the logo should look and what color and shape would represent the cervix in a culturally appropriate way. The study’s logo is shown in Figure 7.

**Figure 8:**
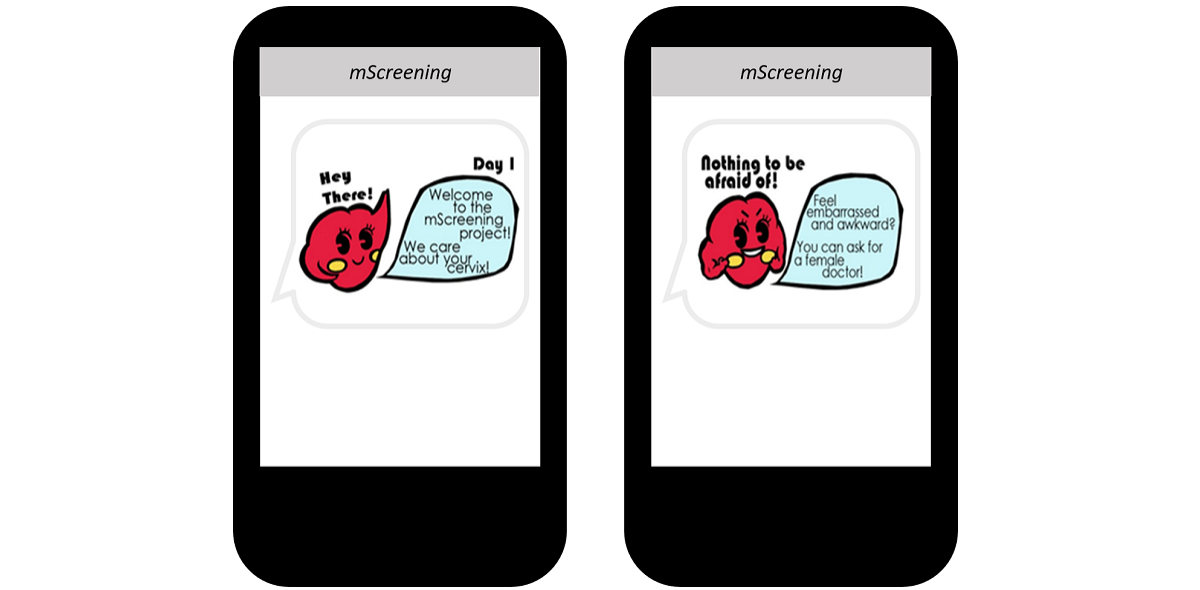
Mobile screening message example: developed multiple characters to help target population feel more positively engaged with the intervention.

With input from focus group participants and community advisory board members, we revised the logo and developed multiple characters depicting various facial expressions and used them in mScreening messages to help the target population feel more positively engaged with the intervention and at ease with the information presented (Figure 8).

**Figure 9:**
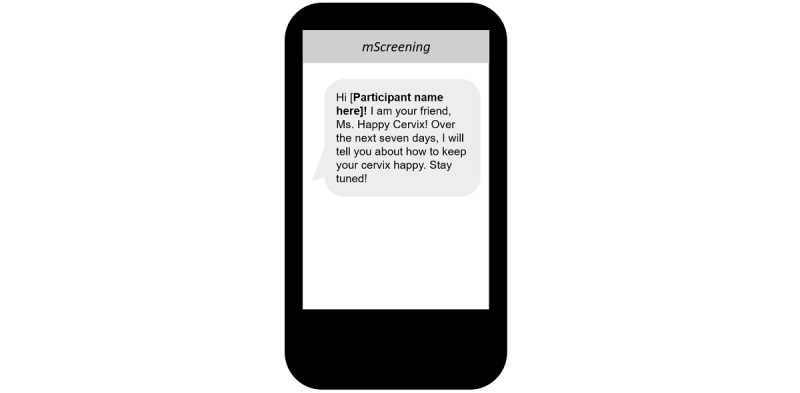
Mobile screening message example: tailored welcoming message that addressed the participant by her first name or nickname.

In addition, the first text of each day was a tailored, welcoming message that addressed the participant by her first name or nickname (Figure 9).

##### Subtheme 2.3: Interactive Messages

Participants suggested that interactive messages involving question and answer (Q&A) or games should be included in the intervention to minimize barriers of a 1-way text messaging delivery system. Participants expressed wanting the ability to interact with others involved in the project or the content provider so that they would able to communicate their questions or any confusion they had. Participants also pointed out some benefits of Q&A format messages:

It would be good to mix information and questions. How about we receive related information when we answer a question? In this way, messages can be tailored for each individual and this would increase study engagement.

Participant #18

**Figure 10:**
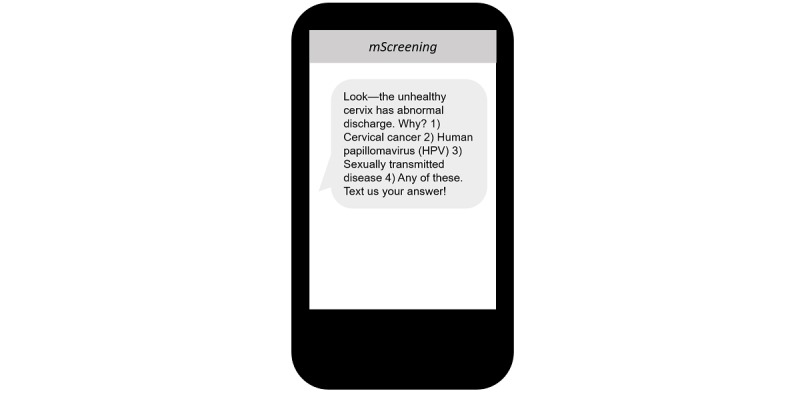
Mobile screening message: developed interactive question and answer messages.

To ensure the inclusion of interactive messages with the intent to promote participants’ attentiveness and motivation during the intervention, 10 interactive Q&A messages were developed (Figure 10).

If a participant texted answer (1), for example, then an appropriate response message was sent to the participant so that the tailored message was relevant for the participant. A gynecologic doctor who served on the community advisory board and worked at the University Health Center reviewed all the texts for medical accuracy and confirmed that all text message content used was accurate. We also had a bilingual health navigator, who is a registered nurse, available for questions and comments as part of *mScreening.* We provided the phone number of the health navigator each day at the end of the intervention and asked if each participant had a question or concern to discuss with the health navigator.

#### Theme 3: Message Delivery Formats

Questions centering on how to deliver suggested content were also discussed in the focus groups. Participants were asked about their preferred length, frequency, interval between messages, and duration of messages, as well as potentially appealing program names. Participants suggested that short and concise messages with online resources should be included, given that short message service text messages are generally limited to 160 characters maximum for a regular mobile phone. As 1 participant indicated:

A message through a regular mobile phone is limited to 160 characters. It could be hard to provide information in detail. What about sending a short message with a Facebook or website link where we can explore information further as needed?

Participant #2

**Figure 11:**
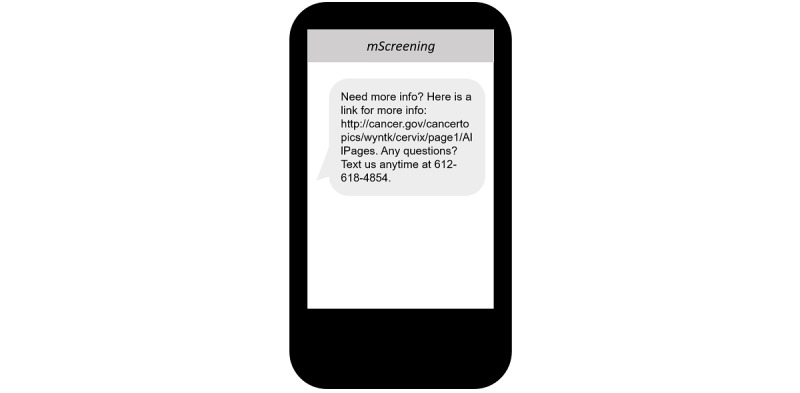
Mobile screening message example: provided one message that contained an online link for further information.

To address the limit on the length of text messages, succinct messages were created and incorporated into *mScreening.* In addition, on the last day of the intervention, participants were provided with 1 message that contained an online link for further information (Figure 11).

In terms of the number of messages received and the duration of the intervention, approximately 3 messages sent daily from *mScreening* over the course of a week to a month was considered acceptable. The participants also expressed that they wanted to be asked about their preferred time of day for receiving messages before the program started. After discussion with community advisory board members and mobile phone technology experts, the research team decided to develop a 1-week program with a more intensive frequency of messages and assess participants’ preferred time for message delivery through the pretest questionnaire. More specifically, based on focus group participants’ input, *mScreening* was designed to deliver each participant 15 to 20 messages every day, depending on each day’s topic, over the course of 7 days. One concern was that messages to their mobile phones would message their spam instead. Participants suggested that creating an appealing name for the intervention would help the target population recognize the messages as coming from a welcome source:

It might be better to have a program name such as “big sister.” I prefer to have the feeling that a sister (who has known me for a while) takes care of me and gives me that kind of information. A sister sounds friendlier than a mother or doctor.

Participant #5

The research team used *mScreenin* g as the intervention name, which indicates a mobile phone–based cervical cancer screening and prevention program. The intervention was designed to ensure that a welcoming message incorporating the name would be sent as the first message each day to prevent the participants from confusing the intervention with any potential spam messages.

#### Theme 4: Use of an Incentive for Study Engagement

Another theme present in the focus groups was that of possible remuneration. Participants recommended providing an incentive to maintain high levels of engagement in the intervention. In addition, the participants endorsed the belief that an incentive would help encourage advertisement among participants, leading to the recruitment of more potential participants. In terms of the type of incentive, the participants discussed several possibilities, such as gift cards for coffee or small gifts such as cell phone attachments (which are a trend among young Korean American women). Participants also suggested sending a celebratory message to intervention participants who received a Pap test or a dose of the HPV vaccine after their participation in *mScreening.* The research team created a cell phone attachment with a mini mirror that features the study’s logo. This attachment was provided to participants upon completion of the intervention, whereas a celebratory message was sent after participants received a Pap test or dose of the HPV vaccine.

## Discussion

### Principal Findings

This study sought to demonstrate how a culturally and individually tailored mobile text messaging intervention program, *mScreening,* was developed to promote the uptake of Pap testing and HPV vaccination in young Korean American immigrant women. The message contents, format, and delivery format of *mScreening* were suggested by participants through a series of focus groups. And then, community advisory board members and mobile technology experts reviewed the initial program and refined for intervention use. We identified 4 themes and we used them to inform the development of *mScreening.*

### Comparison With Previous Work

The first theme centers on developing and delivering culturally targeted and tailored messages. Through the use of focus groups, various types of culturally tailored motivating messages were suggested and created to help increase basic knowledge and awareness about cervical cancer, Pap testing, and HPV vaccination. Participants in focus groups suggested that the presentation of information might be sometimes shocking or emotionally affecting in ways consistent with a previous study that promoted sexual health for young adults in Australia [35]; in this study, fear and the use of grave statistics were recommended by young participants as especially effective strategies. A recent study that used guilt-fear appeal for HPV vaccination reported that fear is a mediator between perceived susceptibility and HPV intentions [35].

At the same time, participants suggested that the *mScreening* messages should highlight the benefits of Pap testing and HPV vaccine uptake by framing the messages in terms of potential gain [31]. The recommendation for this gain-framed messaging departs from previous studies that have employed messages framed in terms of potential losses. For example, 1 study suggested that loss-framed cancer screening messages (eg, the costs of not undergoing screening) are more effective and powerful at promoting screening than gain-framed messages (eg, benefits of undergoing screening) would be [31]. The study similarly found that loss-framed messages were more persuasive at promoting HPV vaccination [36]. Other studies have noted that cultural contexts should be considered when messages are framed [37,38]. These studies suggest that message framing alone without consideration of individuals’ cultural characteristics will not be effective. On the basis of these perspectives, we decided to use both gain and loss frames in developing targeted and tailored messages in our study.

As one of the subthemes in Theme 1, culturally tailored content delivery to improve literacy related to health care access was suggested. In addition to culturally tailored information regarding the Pap test and HPV vaccine, *mScreening* messages should deliver practical information on participants’ health insurance coverage and information on physicians and clinics to help enhance health care access. The participants’ suggestions further implied that increasing familiarity with the US health care system might facilitate young Korean American immigrant women’s participation in cervical cancer screening and prevention behavior. This finding reflects the findings from a previous study with Vietnamese American women that demonstrated that access to health care for cancer screening is improved with health navigator service [14]. On the basis of this suggestion, we added a bilingual Korean health navigator service into the *mScreening* intervention so that any barriers to health care service access can be minimized, which may ultimately enhance health care accessibility for cervical cancer screening and HPV vaccine promotion.

The second and third themes focused on an interactive and visually appealing message development. When participants were asked about message formatting, they suggested various methods to help increase study participants’ attention and engagement. Reflecting participant suggestions, the research team developed various types of messages that included images and emoticons, as well as interactive Q&A messages. Previous studies provide supporting evidence that message formats could potentially enhance people’s understanding of information and create a relaxed atmosphere that would also help maintain engagement [39]. For example, as emoticons can be used to express nonverbal communication, such as humor, solidarity, support, positive feelings, and appreciation [39], messages that include emoticons may help create a comfortable atmosphere in which young Korean American immigrant women may feel more willing to explore cervical cancer and talk more easily to peers about their health. In addition, the Q&A messages address the shortcoming of a 1-way text messaging system.

Regarding message delivery format, participants agreed that short and concise messages were preferable, although this could potentially impact the accuracy of the messages. To deliver accurate information despite the short messages, the research team created messages that would lead participants to a reliable online resource for further information. The last theme is about retention strategy for the 3-month intervention period. Participants suggested using an incentive system to maintain high levels of participation, engagement, and retention. Previous studies also reported effectiveness of monetary incentives (eg, gift cards or cash bills) in recruiting potential participants, participant engagement, and reduction of attrition [40,41]. Similarly, a review conducted by Tishler and Bartholomae [42] indicated that financial rewards could play a critical role in the motivation levels for volunteers deciding to participate in research studies.

### Limitations

Although this study has yielded various pieces of critical information, there are several limitations. First, given that the study participants were all young Korean American immigrant women residing in the upper Midwest, the findings cannot be generalized to Korean American immigrant women in other locations at large. Furthermore, although we made an effort to recruit Korean American immigrant women residing in the community, all participants were current college or graduate students who had resided in the United States for brief periods of time, further limiting generalizability of the findings. In recent years, however, the population of Asian American immigrants has raised, including college students and college graduates [43]. As most of participants were fairly new to the United States, English proficiency functioned as a barrier to understand the actual meaning of the text messages. Some of the participants suggested to provide the texts in both Korean and English by providing a language choice. Although they are in college and graduate schools, when it comes to health information, understanding via mother tongue makes them accurately understand each health-related message. Future researchers should further explore the impact of language and importance of tailoring interventions according to participants’ health literacy and acculturation factors. Finally, it is important to mention that the age range of focus group participants was 21 to 29 years—with an average age of 26 years—which is the upper age limit for obtaining the HPV vaccine in girls and women according to age guidelines (9 to 26 for girls and women). In general, the HPV vaccine is promoted to the younger adolescent girls aged 11 to 17 years, even as young as 9 years; thus, the messaging from this study may not be generalizable to the common populations targeted with HPV vaccine messaging.

### Conclusions

Despite the limitations, this study provides critical information in developing culturally targeted and individually tailored motivational messages to bring about positive health behavior change. This study sought to provide guidelines and suggestions to researchers interested in developing text message–based interventions for underserved minority populations. The results of the study highlighted creating and delivering culturally tailored and targeted messages, the use of intervention tools such as visual images and graphic pictures as an effective way of delivering content on sensitive topics, and provision of navigation services for study participants who are immigrants. Furthermore, the participants expressed preferences for interactive messages over 1-way communication that primarily seeks to increase participants’ engagement in the study. In addition, the desired format of messages that seek to improve knowledge of cervical cancer are short and precise messages that provide links to reliable online resources. To the best of the authors’ knowledge, this study is the first that provides information on the process of gathering culturally relevant information for the purpose of designing a mobile phone text messaging intervention to promote Pap tests and HPV vaccines. The study also provided information on the process of developing the intervention based on the target population’s perspectives. Given that mobile phones are not only becoming the most common means of communication among young people but also that mHealth technology has demonstrated its effectiveness as a useful tool for behavioral changes in disease prevention and self-management in chronic diseases [44-50], the findings of this study could potentially guide future development of cancer screening interventions to promote Pap testing and HPV vaccination for other underserved minority groups and other types of cancer screening, including breast, colorectal, and lung cancer.

## Abbreviations

FBM: Fogg behavior model
HPV: human papillomavirus
mHealth: mobile health
mScreening: mobile screening
Pap: Papanicolaou
Q&A: question and answer
